# Commentary: Metaphor in nursing research – a tool for meaning-making, reflection and critical engagement

**DOI:** 10.1177/17449871261452623

**Published:** 2026-06-11

**Authors:** Sarah Bekaert

**Affiliations:** Oxford School of Nursing and Midwifery, Oxford Brookes University, Oxford, UK

**Commentary to:** Metaphorically speaking: considerations in applying metaphor to your research higher degree (Byrne and Mulvogue, 2026).

## Summary of the paper

This paper explores how metaphor can be used as a creative, analytical and pedagogical tool in qualitative research, particularly for research higher degree candidates. Byrne and Mulgrove (2026) argue that metaphor plays a central role in shaping understanding, organising ideas and communicating complex concepts. Metaphors allow researchers to connect abstract ideas with familiar experiences, making research more accessible and meaningful.

The paper presents an eight-step framework to guide the intentional use of metaphor. These steps are:

1. Why use a metaphor? – Clarifying the purpose and value of metaphor in the research.
2. Finding the right metaphor – Selecting a metaphor that fits the meaning, emotion and message of the study.
3. Metaphor to express power and ideology – Using metaphor to reveal or critique power structures and underlying assumptions.
4. Metaphoric imagery – Incorporating visual or descriptive imagery to deepen understanding.
5. Sowing a common thread – Using metaphor to link ideas and create coherence across the research.
6. Metaphor as a method of signposting – Guiding the reader through the work using consistent metaphorical cues.
7. Metaphor as a culmination and catharsis – Using metaphor to bring the research to a meaningful conclusion.
8. Implications – Reflecting on the risks and benefits, including whether metaphor enhances or distorts understanding.

Through worked examples, the paper demonstrates how metaphor can create a coherent ‘thread’ across a body of work. The key message is that metaphor supports deeper thinking and learning. It helps both the researcher and the reader engage with complex, abstract and often emotionally charged topics. This is particularly relevant in nursing research, where experiences are multifaceted and not always easily captured through technical language alone.

## Connection to nursing theory, practice and research

The paper aligns strongly with the nature of nursing as both a scientific and humanistic discipline. Nursing practice involves interpreting and responding to complex human experiences such as pain and vulnerability. Metaphor is embedded in everyday nursing language, with patients and professionals often describing illness as a ‘journey’, the treatment experience as a ‘battle’ and recovery as ‘building strength’ to name a few examples. Byrne and Mulvogue (2026) reinforced that such language is not incidental but can be used intentionally to deepen understanding and communication. For example, Saatçi and Akın (2025) used metaphorical analysis to explore nurses’ perceptions of missed care, identifying multiple thematic categories that revealed the emotional, systemic and risk-related dimensions of practice. This demonstrates how metaphor can uncover aspects of care that may otherwise remain hidden within traditional forms of analysis. Such a perspective connects with established nursing and educational theories. Metaphor can support progression along Benner’s model of skill acquisition, helping learners move from concrete descriptions of care to more abstract, intuitive understanding (Benner, 1984). It also aligns with Carper’s patterns of knowing, particularly aesthetic and personal knowing, where metaphor enables expression of experiences that cannot be fully captured through empirical data alone (Carper, 1978).

The paper also reflects principles of reflective practice, as described by Schön (2017), where metaphor can support both reflection-in-action and reflection-on-action. Contemporary perspectives suggest that reflection in nursing extends beyond process to the development of reflective knowledge and critical understanding (Schutz, 2026). Within this context, metaphor can function as an interpretive tool, supporting deeper engagement with practice by enabling nurses to explore and communicate the complexity, uncertainty and emotional dimensions of care.

In addition, the emphasis on meaning-making and communication aligns with person-centred care approaches, which prioritise understanding the patient’s lived experience and narrative (McCance and McCormack, 2025). However, this ideal is not always realised in practice, as there are tensions in providing person-centred nursing care within complex healthcare environments (Byrne, 2025). This tension is particularly relevant to the use of metaphor in research, as metaphor may provide a way of articulating the gap between policy ideals and the realities of clinical practice. By translating complex organisational and relational dynamics into more accessible forms, metaphor can support critical reflection and make visible the constraints that shape nursing care. Overall, the paper’s findings are consistent with observations in practice. It reinforces what is often done intuitively in nursing, using metaphor to explain, reflect and connect but provides a structured way to apply this more critically and effectively in research (e.g. in Saatçi and Akın 2025).

## Practical implications: who can benefit from this research?

This paper is particularly valuable for nursing students, doctoral candidates, clinical researchers, educators and supervisors. It provides a clear and practical framework that can be applied across research, teaching and practice.

In education, metaphor can support learning by enabling students to engage with complex theories and abstract concepts in more accessible ways. It can also enhance reflective writing and critical thinking by providing a means to interpret and articulate experience. For research students, the framework offers a way to create coherence across a thesis, linking different components into a unified and meaningful narrative. This is particularly valuable in qualitative research, where findings are often interpretive, nuanced and multifaceted. Such applications are evident in qualitative nursing research, where metaphor is embedded within both the design and presentation of studies. For example, Jestico (2024) employed an extended journey metaphor to structure a phenomenological exploration of parents’ experiences, using narrative, imagery and music to evoke meaning and support interpretation. This demonstrates that metaphor can function not only as a communicative device but also as an organising and interpretive framework within research.

In clinical practice, metaphor can support communication with patients and families, particularly when discussing complex or sensitive issues. It can also enhance team communication by providing shared ways of understanding challenging situations. However, Byrne and Mulgrove (2026) appropriately highlighted that metaphor must be used carefully. Poorly chosen metaphors may oversimplify, misrepresent or impose meaning on patients’ experiences, which has ethical implications in nursing practice.

The insights are particularly relevant in areas such as mental health, palliative care, children’s nursing and public health, where emotional, social and contextual factors are central. In these settings, metaphor can help make invisible or abstract experiences more visible and understandable.

Overall, the paper demonstrates that metaphor is a valuable tool for bridging the gap between theory, research and practice. When used thoughtfully, it can enhance communication, support critical thinking and strengthen the impact of nursing research.

## References

[bibr1-17449871261452623] Benner P (1984) From novice to expert excellence and power in clinical nursing practice. AJN The American Journal of Nursing 84: 1479.

[bibr2-17449871261452623] Byrne A-L Mulvogue J (2026) Metaphorically speaking: considerations in applying metaphor to your research higher degree. Journal of Research in Nursing. Epub ahead of print 20 January, 2026. DOI: 10.1177/17449871251403373 PMC12823363 41585317

[bibr3-17449871261452623] Byrne A-L (2025) Two sides of the same coin: Person-centred systems versus person-centred nursing practice. Theory, barriers and opportunities. Journal of Research in Nursing 30: 6–17. DOI: 10.1177/17449871241255012. 40255929 PMC12003329

[bibr4-17449871261452623] Carper BA (1978) Fundamental patterns of knowing in nursing. Advances in Nursing Science 1: 13–24. 110216 10.1097/00012272-197810000-00004

[bibr5-17449871261452623] Jestico E (2024) Shaping Confidence to Advocate for My Child: A Hermeneutic Phenomenological study. Oxford: Oxford Brookes University.

[bibr6-17449871261452623] McCance T McCormack B (2025) The person-centred nursing framework: A mid-range theory for nursing practice. Journal of Research in Nursing 30: 47–60. DOI: 10.1177/17449871241281428. 40093819 PMC11907491

[bibr7-17449871261452623] Saatçi G Akın E (2025) Decoding missed nursing care: The power of metaphorical analysis. Journal of Research in Nursing 0(0). DOI: 10.1177/17449871251380437. PMC12626848 41268121

[bibr8-17449871261452623] Schön DA (2017) The Reflective Practitioner: How Professionals Think in Action. London: Routledge.

[bibr9-17449871261452623] Schutz S (2026) Reflection, reflective practice and reflective knowledge in nursing: Where are we? A perspective. Journal of Research in Nursing 17449871251407833. 10.1177/17449871251407833 PMC12846895 41613247

